# Characterization of Clinical and Histopathological Profiles of Oral Potentially Malignant Disorders With Oral Epithelial Dysplasia: Focus on Subepithelial Lymphoplasmacytic Infiltrate and Its Grading

**DOI:** 10.1002/cre2.70482

**Published:** 2026-10-11

**Authors:** Abbas Saleem Khan, Shahzad Ahmad, Hina Shabir, Shazia Iqbal, Tehmina Naushin, Muhammad Nadeem Baig, Aneela Bashir, Maria Hanif, Mohammed Saad Alqarni, Rakhi Issrani

**Affiliations:** ^1^ Department of Oral Pathology, Peshawar Dental College Riphah International University Islamabad Pakistan; ^2^ Faculty of Medicine and Health Sciences The University of Buckingham Buckingham UK; ^3^ Department of Oral Pathology Khyber College of Dentistry Peshawar Pakistan; ^4^ Department of Preventive Dentistry, College of Dentistry Jouf University Sakaka Kingdom of Saudi Arabia; ^5^ Department of Oral Pathology Abbotabad International Medical Institute Abbotabad Pakistan; ^6^ Rashid Latif Dental College Lahore Pakistan; ^7^ Department of Oral & Maxillofacial Surgery and Diagnostic Sciences, College of Dentistry Jouf University Sakaka Kingdom of Saudi Arabia; ^8^ Department of Research Analytics, Saveetha Dental College and Hospitals, Saveetha Institute of Medical and Technical Sciences Saveetha University Chennai India

**Keywords:** grading systems, lymphoplasmacytic infiltrate, oral epithelial dysplasia, oral potentially malignant disorders, oral squamous cell carcinoma

## Abstract

**Objectives:**

This study was conducted to describe the clinical and histopathological features of OPMDs characterized by OED with a special focus on the subepithelial LPI and its grades.

**Material and Methods:**

This cross‐sectional study was conducted on hematoxylin and eosin‐stained slides of 82 diagnosed cases of OPMDs with OED. Histologic review was performed based on the WHO and binary grading system for OED. Subepithelial LPI and its grades were also noted and reported. *χ* ^2^ test and Fisher's exact test were used to compare different categorical variables related to OPMDs.

**Results:**

The mean age of OPMD cases with OED was 58.51 ± 12.38, with a male‐to‐female ratio of 2.28:1. OED lesions were mostly (*N* = 41; 50%) seen on the buccal mucosa, retromolar area, and vestibule of the oral cavity. Oral leukoplakia was the most (*N* = 52; 63.4%) commonly diagnosed clinical variant of OPMDs. Drop‐shaped rete ridges (*N* = 78; 95.12%) and hyperchromasia (*N* = 76; 92.68%) were the most commonly occurring architectural and cellular alterations of OED, respectively. The overall interobserver agreement for various histopathologic variables ranged from moderate to perfect. Histologically, moderate dysplasia was the most frequently occurring grade (*N* = 37; 45.12%). Most (*N* = 45; 54.9%) of the OPMDs were marked as high‐risk dysplasia. Subepithelial LPI was significantly (*p* < 0.01) related to the histologic grades of OED lesions. The grades of LPI were significantly (*p* = 0.018) related to the binary grading system only.

**Conclusion:**

The subgrades of LPI were significantly associated with the binary grading system of OED, suggesting that subepithelial LPI may reflect underlying microenvironmental changes related to dysplasia severity.

## 1 Introduction

Oral potentially malignant disorders (OPMDs) are defined as “any oral mucosal abnormality that is associated with a statistically increased risk of developing oral cancer” (Warnakulasuriya 2020). They are chronic and progressive epithelial disorders which are precursors to oral squamous cell carcinoma (OSCC) (Muller and Tilakaratne 2023). The reported prevalence of OPMD varies markedly among individual studies, hospital‐ and community‐based surveys, populations with different compositions and patterns of tobacco usage, data collection tools, countries, and developed nations and the developing world. However, the agreed prevalence rate lies between 1% and 5% (Warnakulasuriya 2020; Muller and Tilakaratne 2023; Villa et al. 2025). The etiology of OPMDs is multifactorial, and numerous risk factors have been reported to be involved in the development of these lesions. Among them, oral tobacco use and alcohol consumption are considered established risk factors (Sari et al. 2024). Characteristically, OPMDs present with diverse clinical attributes, such as color variations (white, red, and mixed white‐red), morphological changes (plaque/plateau, smooth, grooved, wrinkled, granular, atrophic), and different sizes, involving different anatomical sites in the oral cavity (Warnakulasuriya 2020; Muller and Tilakaratne 2023; Sari et al. 2024; Menditti et al. 2024). OPMDs may be characterized histopathologically as epithelial hyperplasia (hyperkeratosis, hyperparakeratosis, hyperorthokeratosis, acanthosis, and basilar hyperplasia), epithelial atrophy, oral epithelial dysplasia [OED] (mild, moderate, severe dysplasia (Parakh et al. 2020; Mahmood et al. 2022). The gold standard for assessing architectural and cytological alterations not only predicts the histologic grade but also provides insight into the probable prognostic significance of the OED lesion (Mahmood et al. 2022). Histopathologically, OPMDs with oral OED are considered likely precursors to malignant transformation (de Azevedo et al. 2021). A process characterizes malignant transformation by which nonmalignant or potentially malignant cells acquire the characteristics of malignancy due to the aggregation of multiple genetic changes involved in the initiation and progression of malignant tumor. Although the biological mechanisms underlying the malignant transformation of OED into OSCC are not fully understood, various prognostic factors have been identified. A meta‐analysis of cases with OED observed a malignant transformation rate of 12%. Also, moderate to severe forms of OED are more commonly associated with malignant transformation (Villa et al. 2025; Mahmood et al. 2022; Iocca et al. 2020). The overall malignant transformation rate of OPMDs is 7.9%. However, the malignant transformation rate estimated for proliferative verrucous leukoplakia and oral erythroplakia is considerably higher (Warnakulasuriya 2020; Sari et al. 2024; Iocca et al. 2020; Celentano and Cirillo 2024).

OPMDs are lesions with an increased risk of malignant transformation; however, not all OPMDs progress to OSCC. Their progression depends on multiple genetic, environmental, and microenvironmental factors, including inflammatory changes within the tumor microenvironment (Mahmood et al. 2022; Iocca et al. 2020; Speight et al. 2018). The tissue microenvironment of OPMDs appears to play a significant role in their potential to acquire the characteristics of oral epithelial malignancy. The tissue microenvironment of OED includes epithelial cells, the underlying subjacent connective tissue, and inflammatory immune cells (Deng et al. 2022; Rangel et al. 2022).

Regarding OPMDs, the assessment of histologic grading is still a gold standard for prognostic prediction and management planning (Warnakulasuriya 2020; Kujan et al. 2006). The present study also assessed certain micro‐environmental alterations, such as subepithelial lymphoplasmacytic infiltrate (LPI), and its relationship with the histologic variables of OPMDs, specifically with OED. We hypothesized that the tissue microenvironment of OED lesions plays a significant role in their potential to acquire the characteristics of oral epithelial malignancy. Limited studies have explored the relationship between the binary grading system of OED and the degree of subepithelial LPI (Kujan et al. 2006; Halter and Dellon 1983). Therefore, the present study aimed to evaluate this association using both the WHO classification (5th edition, 2023) (Muller and Tilakaratne 2023) and the binary grading system (Kujan et al. 2006).

## 2 Materials and Methods

This descriptive cross‐sectional study was conducted using the data records of 82 histopathologically diagnosed cases of OPMDs with OED. These biopsies were assessed at the Department of Oral Pathology, Peshawar Dental College, Riphah International University, Pakistan, from April 2016 to December 2022. Ethical approval for the study was obtained from the Institutional Review Board of Prime Foundation, Pakistan. The work was conducted in accordance with the Code of Ethics of the World Medical Association (Declaration of Helsinki) (de Azevedo et al. 2021).

### 2.1 Inclusion and Exclusion Criteria

Oral mucosal lesions clinically characterized by white, red, or mixed white/red coloration and histopathologically diagnosed as OPMD with OED were included. Cases with oral epithelial hyperplasia only or OED with coexisting histologic signs of anaplasia and microinvasive/invasive squamous cell carcinoma, as documented in the biopsy report, were excluded.

### 2.2 Data Collection

A convenience nonprobability sampling technique was used. Data collected included demographic variables (age, gender), clinical characteristics (type and site of OPMDs), histopathological features (architectural and cytological changes), grading of OED (WHO and binary systems), and subepithelial LPI and its grades. Hematoxylin and eosin‐stained slides of the OPMD cases with OED were retrieved from the archives of the mentioned departments and reassessed histopathologically using a light microscope.

### 2.3 Extent of Observer Agreement

Three observers independently evaluated the slides: two oral histopathologists (H. S. and T. N.) with experience in grading OPMDs and OSCC, and one histopathologist (A. S. K.) with dual training in general and oral pathology. The observers were blinded to the initial diagnosis and clinical information. Each slide was marked with a specific number. Calibration sessions were jointly conducted by all three observers prior to slide evaluation to standardize interpretation criteria. A score sheet for the cytological and architectural findings was designed for recording the histopathologic details of the cases to ensure standardization of reporting. Each observer was asked to grade each case according to the WHO Classification of Head and Neck Tumors (5th edition, 2023) (Muller and Tilakaratne 2023) and the binary grading system proposed by Kujan et al. (2006). The slide assessment was repeated, with the same events described, after 2 weeks, and the slides were relabeled to avoid bias for the second round of histopathologic assessment. While assessing the interobserver variability, the examiner agreement levels demonstrated in the study were 0.927 (perfect agreement) for the WHO grading system of OED, 0.730 (substantial agreement) for the binary grading system of OED, 0.859 (almost perfect agreement) for LPI, and 0.153 (slight agreement) for the newly proposed grades of LPI.

### 2.4 Evaluation of OED

The classification system for OED lesions by WHO was used to categorize OPMDs with dysplastic alterations (Muller and Tilakaratne 2023). According to this system, OPMDs with OED were classified as mild dysplasia, moderate dysplasia, and severe dysplasia. OED was assessed based on architectural alterations (e.g., irregular epithelial stratification, loss of polarity of basal cells, drop‐shaped rete ridges, increased number of mitotic figures, abnormally superficial mitotic figures, keratin pearls within rete ridges, and loss of epithelial cohesion) and cytological alterations (e.g., abnormal variation in cell and nuclear size and shape, increased nucleus‐to‐cytoplasm ratio, atypical mitotic figures, increased number and size of nucleoli, premature keratinization in single cells, and hyperchromasia) (Mahmood et al. 2022). A binary system of OED was also used, with cutoff points for four architectural and five cytological features to classify OED as high‐risk dysplasia (with potential for malignant transformation) or low‐risk dysplasia (without potential for malignant transformation) lesions (Mahmood et al. 2022; de Azevedo et al. 2021).

### 2.5 Evaluation of Subepithelial LPI

The degree of subepithelial LPI in the histologic sections of OPMDs with OED, stained with H&E, was graded by initially scanning the specimen slide at low power magnification and then assessing at ×40 magnification to subjectively appreciate and grade the plasma cell and lymphocytic cell populations. Plasma cells were defined as infiltrating cells with an eccentric nucleus and a prominent rim of basophilic cytoplasm. LPI was graded as – marked (+++) when LPI presented as a continuous rim at the epithelia–mesenchymal junction, moderate (++) when LPI was observed as large patches at the epithelia–mesenchymal junction, slight (+) when a few occasional patches of LPI were observed, and (−) indicating absence of LPI in the observed sections (Halter and Dellon 1983).

### 2.6 Statistical Analysis

Data were tabulated descriptively, with variables related to the clinic‐pathologic features of OPMDs with OED. Results are presented as means and standard deviations for age and gender. *χ* ^2^ tests and Fisher's exact tests were used to compare different categorical variables related to OPMDs. Inter‐rater reliability was evaluated by Cohen's *κ* statistics. Statistical analysis was performed using Statistical Package for the Social Sciences software version 20.0 (IBM Corp., Armonk, NY, USA), with a significance level of *p* ≤ 0.05.

## 3 Results

The recorded ages of OPMD cases with OED ranged from 20 to 80 years, with a mean age of 58.51 ± 12.38 years, a median age of 60 years, and an interquartile range of 19 years (48–67 years). The mean age for males was 59.51 (SD = 11.96; range: 35–82 years), while for females it was 56.16 (SD = 13.26; range: 20–74 years). Among the patient cohort, 57 (69.5%) were male, resulting in a male‐to‐female ratio of 2.28:1. Most cases of OED were observed in older individuals. High‐risk lesions were more commonly observed in older patients. A significant relationship was found between the binary grading system of OED lesions and the age of OPMD cases (Table 1).

**Table 1:** Demographic characteristics of study population in relation to WHO and binary grading systems of OED (*N* = 82).

Study variables	WHO grading: mild dysplasia (*N* = 26)	WHO grading: moderate dysplasia (*N* = 37)	WHO grading: severe dysplasia (*N* = 19)	Binary grading: low‐risk dysplasia (*N* = 37)	Binary grading: high‐risk dysplasia (*N* = 45)	Total
	*N* (%)	*N* (%)	*N* (%)	*N* (%)	*N* (%)	*N* (%)
*Age range in years*						
20–40	3 (3.7%)	2 (2.4%)	0 (0.0%)	4 (4.9)	1 (1.2)	5 (6.1%)
41–50	5 (6.1%)	8 (9.8%)	8 (9.8%)	9 (11.0)	12 (14.6)	21 (25.6%)
51–60	3 (3.7%)	10 (12.2%)	6 (7.3%)	4 (4.9)	15(18.3)	19 (23.2%)
> 60	15 (18.3%)	17 (20.7%)	5 (6.1%)	20 (24.4)	17 (20.7)	37 (45.1%)
	*p* = 0.131*			*p* = 0.043*		—
Male	21 (25.6%)	24 (29.3%)	12 (14.6%)	29 (35.4)	28 (34.1)	57 (69.5%)
Female	5 (6.1%)	13 (15.9%)	7 (8.5%)	8 (9.8)	17 (20.7)	25 (30.5%)
	*p* = 0.318*			*p* = 0.114*		—

*Note:* Data are presented as frequency and percentage, *N* (%). WHO grading categorizes lesions as mild dysplasia, moderate dysplasia, and severe dysplasia whereas the binary grading system categorizes lesions as low‐risk and high‐risk dysplasia.

* Pearson's *χ* ^2^ test.

When assessing cellular and architectural alterations in OED lesions, the most frequently observed cellular feature was “hyperchromasia” (*N* = 76; 92.68%), while the most common architectural change was “drop‐shaped rete ridges” (*N* = 78; 95.12%) among OPMD cases with OED (Table 2). Figure 1A shows a photomicrograph of a severe OED lesion in the buccal mucosa, characterized by prominent drop‐shaped rete ridges and a cenctral area of dense lymphoplasmacytic inflammatory infiltrate in the underlying connective tissue, magnified at ×10.

**Table 2:** Frequency of the observed cellular and architectural alterations in oral epithelial dysplastic lesions (*N* = 82).

Cellular changes in OED lesions	Frequency *N* (%)	Architectural changes in OED lesions	Frequency *N* (%)
Anisonucleosis	40 (48.78%)	Irregular epithelial stratifications	68 (82.92%)
Nuclear pleomorphism	46 (56.09%)	Loss of polarity of basal cells	51 (62.19%)
Anisocytosis	39 (47.5%)	Drop‐shaped rete ridges	78 (95.12%)
Cellular pleomorphism	75 (91.46%)	Increased no. of mitotic figures	19 (23.17%)
Increased nuclear‐to‐cytoplasmic ratio	49 (59.75%)	Abnormal superficial mitosis	19 (23.17%)
Atypical mitotic figures	19 (23.17%)	Intraepithelial keratin pearls	3 (3.65%)
Increased no. and size of nucleoli	31 (37.80%)	Loss of epithelial cell cohesion	6 (7.31%)
Hyperchromasia	76 (92.68%)		
Dyskeratosis	45 (54.87%)		

*Note:* Data are presented as frequency and percentage, *N* (%).

Abbreviation: OED, oral epithelial dysplasia.

**Figure 1:**
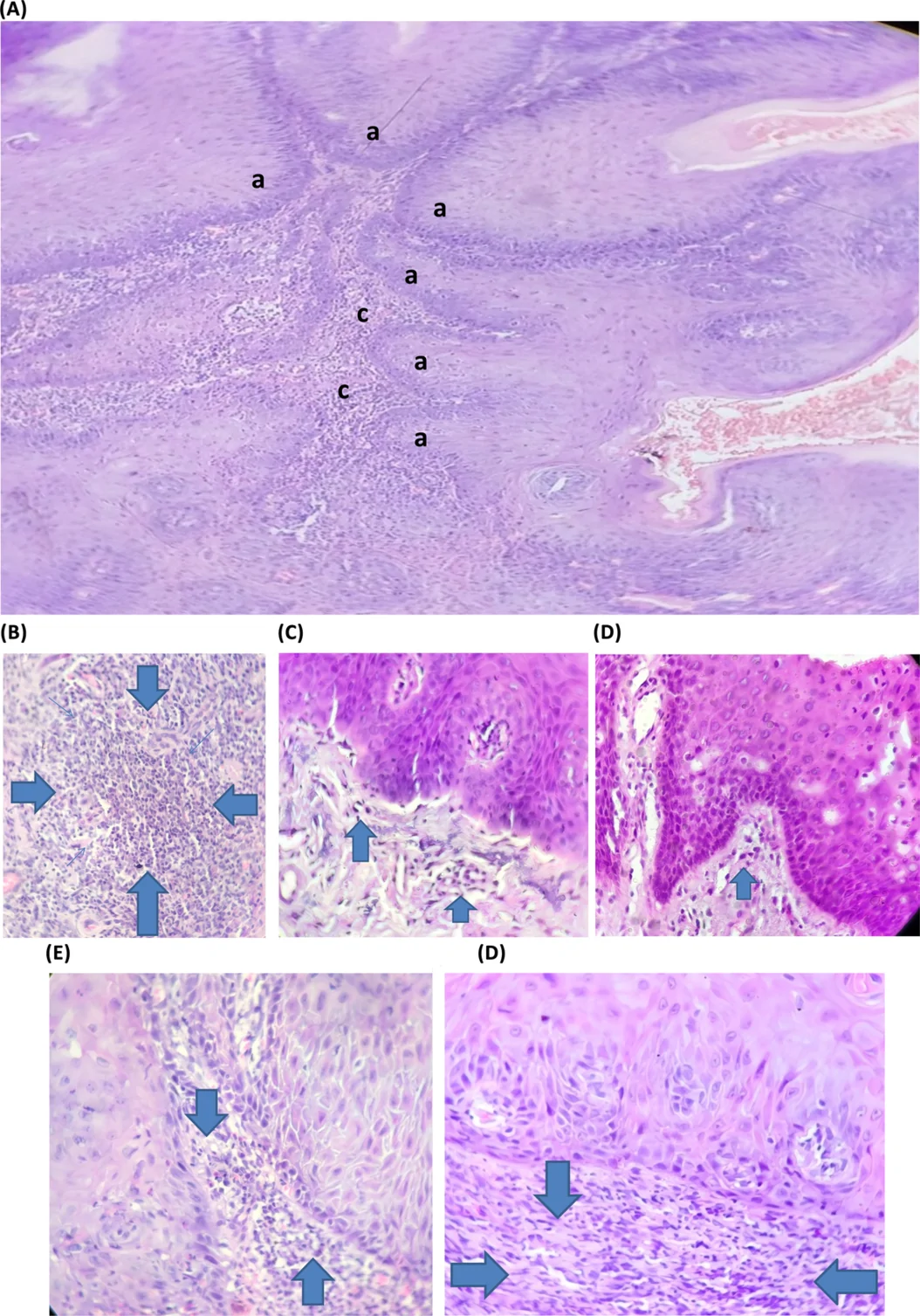
(A–F) Representative high‐resolution photomicrographs of oral epithelial dysplasia and subepithelial lymphoplasmacytic infiltrate (LPI). (A) A photomicrograph of the buccal mucosa demonstrates a severe OED lesion characterized by prominent drop‐shaped rete ridges accompanied by a central area of dense lymphoplasmacytic inflammatory infiltrate in the underlying connective tissue (hematoxylin and eosin (H&E) stain; original, magnification ×10). (B) A photomicrograph of the buccal mucosa showing a dense lymphoplasmacytic infiltrate (H&E stain; original, magnification ×40). (C) A photomicrograph of the floor of the mouth showing the absence of a significant subepithelial lymphoplasmacytic inflammatory infiltrate (H&E stain; original, magnification ×40). (D) A photomicrograph of the gingiva showing a mild lymphoplasmacytic inflammatory infiltrate, present as scattered foci in the subepithelial connective tissue (H&E stain; original, magnification ×40). (E) A photomicrograph of the retromolar trigone showing a moderate (++) lymphoplasmacytic inflammatory infiltrate, in a large patch, with in the subepithelial connective tissue (H&E stain; original, magnification ×40). (F) A photomicrograph of the gingiva showing dense [marked (+++)] band‐like lymphoplasmacytic inflammatory infiltrate is present within the subepithelial connective tissue (H&E stain; original, magnification ×40).

Among OED cases, the most common sites of OPMD lesions were the buccal mucosa, retromolar area, and vestibule of the oral cavity (*N* = 41; 50%). More than half of the cases (*N* = 52; 63.4%) presented clinically with oral leukoplakia. Of the histologically assessed OED lesions, most were classified as high‐risk dysplasia (*N* = 45; 54.9%) according to the binary system of OED grading. Subepithelial LPI was noted in most cases of moderate dysplasia (*N* = 34; 41.4%). Statistically significant relationships were observed between the various histological grades of OED on the basis of WHO grading system, clinical subtypes of OPMDs (*p* = 0.001), risk of lesion (*p* = 0.001), and subepithelial LPI (*p* = 0.013). However, no statistically significant relationship was observed for different grades of subepithelial LPI (*p* = 0.573). Most high‐risk dysplasia developed in the cheek mucosa, vestibule of the mouth, and retromolar area (*N* = 24/45). More than half of the OL lesions were classified as low‐risk dysplasia (*N* = 28/37), while most oral erythroplakic lesions were diagnosed as high‐risk lesions (*N* = 13/14). Subepithelial inflammatory infiltrate was most commonly expressed in high‐risk dysplasia (*N* = 44/62; 53.7%). Statistically significant differences were observed between the binary grading system of OED and the clinical presentation of OPMDs (*p* = 0.004), histologic grade of OED (on the basis of WHO grading system) [*p* = 0.001], LPI (*p* = 0.011), and subgrades of LPI (*p* = 0.018) (Table 3). Figure 1B–F shows representative photomicrographs of LPI and its subgrading at different anatomical sites, including the buccal mucosa (Figure 1B), floor of the mouth (Figure 1C), gingiva (Figure 1D,F), and retromolar trigone (Figure 1E), at ×40 magnification.

**Table 3:** Clinicopathological characteristics of OPMDs in relation to WHO and binary grading systems (*N* = 82).

Variables	WHO grading: mild dysplasia (*N* = 26)	WHO grading: moderate dysplasia (*N* = 37)	WHO grading: severe dysplasia (*N* = 19)	Binary grading: low‐risk dysplasia (*N* = 37)	Binary grading: high‐risk dysplasia (*N* = 45)	Total
	*N* (%)	*N* (%)	*N* (%)	*N* (%)	*N* (%)	*N* (%)
*Site of lesion*						
Tongue	10 (12.2)	9 (11.0)	3 (3.7)	12 (14.6)	10 (12.2)	22 (26.8)
Gingiva	1 (1.2)	3 (3.7)	5 (6.1)	3 (3.7)	6 (7.3)	9 (11.0)
Floor of mouth	0 (0.0)	2 (2.4)	1 (1.2)	1 (1.2)	2 (2.4)	3 (3.7)
Palate	1 (1.2)	2 (2.4)	1 (1.2)	2 (2.4)	2 (2.4)	4 (4.9)
Buccal mucosa/vestibule/retromolar	12 (14.6)	21 (25.6)	8 (9.8)	17 (20.7)	24 (29.3)	41 (50.0)
Lip	2 (2.4)	0 (0.0)	1 (1.2)	2 (2.4)	1 (1.2)	3 (3.7)
	*p* = 0.248*			*p* = 0.808*		—
*Clinical type*						
OL	21 (25.6)	24 (29.3)	7 (8.5)	28 (34.1)	24 (29.3)	52 (63.4)
SL	2 (2.4)	9 (11.0)	1 (1.2)	4 (4.9)	8 (9.8)	12 (14.6)
OLP	1 (1.2)	1 (1.2)	0 (0.0)	2 (2.4)	0 (0.0)	2 (2.4)
OE	1 (1.2)	2 (2.4)	11 (13.4)	1 (1.2)	13 (15.9)	14 (17.1)
PVL	1 (1.2)	1 (1.2)	0 (0.0)	2 (2.4)	0 (0.0)	2 (2.4)
	*p* = 0.001*			*p* = 0.004*		—
*Binary grading vs. WHO*						
Low risk	26 (31.7)	11 (13.4)	0 (0.0)	—	—	37 (45.1)
High risk	0 (0.0)	26 (31.7)	19 (23.2)	—	—	45 (54.9)
	*p* = 0.001*			—	—	—
*LPI (presence)*						
Present	22 (26.9)	34 (41.4)	18 (22.0)	30 (36.6)	44 (53.7)	74 (90.3)
Absent	4 (4.9)	3 (3.7)	1 (1.2)	7 (8.5)	1 (1.2)	8 (9.8)
	*p* = 0.013*			*p* = 0.011^		—
*LPI grades*						
None (−)	4 (4.9)	3 (3.7)	1 (1.2)	7 (8.5)	1 (1.2)	8 (9.8)
Slight (+)	10 (12.2)	14 (17.1)	4 (4.9)	15 (18.3)	13 (15.9)	28 (34.1)
Moderate (++)	9 (11.0)	12 (14.6)	9 (11.0)	11 (13.4)	19 (23.2)	30 (36.6)
Marked (+++)	3 (3.7)	8 (9.8)	5 (6.1)	4 (4.9)	12 (14.6)	16 (19.5)
	*p* = 0.573*			*p* = 0.018*		—

*Note:* Data are presented as frequency and percentage, *n* (%).

Abbreviations: LPI, lymphoplasmacytic inflammatory infiltrate; OE, oral erythroplakia; OED, oral epithelial dysplasia; OL, oral leukoplakia; OLP, oral lichen planus; OPMD, oral potentially malignant disorder; PVL, proliferative verrucous leukoplakia; SL, speckled leukoplakia.

* Pearson's *χ* ^2^ test.

^^^ Fisher's exact test.

## 4 Discussion

The present study provides a comprehensive clinicopathological evaluation of OPMDs with OED by integrating WHO grading, binary grading, and subepithelial LPI. The key findings include: (1) moderate dysplasia as the most common histological grade, (2) a predominance of high‐risk lesions based on the binary system, (3) significant associations between LPI and dysplasia severity, and (4) a novel association between LPI subgrading and the binary grading system. OED lesions are considered to represent various stages of progression from normal oral mucosa to anaplastic oral epithelium. Characterizing OPMDs with OED is crucial as it helps clinicians identify lesions with high and low risk for conversion into OSCC, thereby guiding effective treatment planning. Accurate histologic characterization using various grading systems is essential for developing a constructive treatment plan for OPMDs with OED. This approach not only helps clinicians identify high‐ and low‐risk dysplasia but also improves diagnosis and prognosis of oral epithelial malignancy (Mahmood et al. 2022; Kujan et al. 2006; Saleem Khan et al. 2021; Kumari et al. 2022).

### 4.1 Histopathological Characteristics of OED

The top 5 observations related to cellular alterations in our study were hyperchromasia, cellular pleomorphism, increased nuclear‐cytoplasmic ratio, nuclear pleomorphism, and anisonucleosis. The most prevalent architectural features included drop‐shaped rete ridges, irregular epithelial stratification, loss of basal cell polarity, dyskeratosis, and both abnormal superficial mitosis and increased mitotic figures. These findings align with other research. Câmara et al. reported common cytological alterations as increased number and size of nucleoli, hyperchromatism, nuclear pleomorphism, anisonucleosis, and increased nuclear size, while common epithelial alterations included irregular epithelial stratification, loss of basal cell polarity, and drop‐shaped rete ridges. However, all cases in their study were of actinic cheilitis (Câmara et al. 2016). Similarly, de Azevedo et al. (2021) found increased number and size of nucleoli, loss of basal cell polarity, and variations in cellular size and shape as common features in 953 cases of OED, with basal cell hyperplasia and irregular surface keratin as prevalent histological features, and verrucous surface, loss of epithelial cohesion, lymphocytic band, and dyskeratosis as less common.

### 4.2 Anatomical and Clinical Characteristics of OPMDs

In our study, the anatomical distribution of OED lesions was most commonly reported in the buccal mucosa, vestibule of the mouth, and retromolar area, followed by the tongue and gingiva. Previous studies have also noted similar findings, with dysplastic lesions commonly seen on the lateral border of the tongue, alveolar ridge, and gingiva (Mahmood et al. 2022; de Azevedo et al. 2021; Câmara et al. 2016; Evren et al. 2023; dos Santos Pereira et al. 2011). The tongue and floor of the mouth are strongly associated with an increased risk of progression to oral epithelial malignancy (Speight et al. 2018).

Among OED lesions, a predominance of OL was observed, consistent with other studies (de Azevedo et al. 2021; Speight et al. 2018; Aittiwarapoj et al. 2019). A statistically significant relationship was found between the clinical presentation of OED and the grades of OED and risk of lesion. These findings are consistent with de Azevedo et al. (2021) but contrary to Aittiwarapoj et al. (2019). In a systematic review and meta‐analysis by Mello et al. (2018), the prevalence of OPMDs was reported as follows: oral submucous fibrosis 4.47%, oral leukoplakia 4.1%, actinic cheilosis 2.08%, and oral erythroplakia 0.17%.

### 4.3 Age and Gender Distribution

In our study, the age range for cases was 20–82 years, with a mean age of 58.51 (SD = 12.38). This is consistent with de Azevedo et al. (59.7) (de Azevedo et al. 2021) but contrary to dos Santos Pereira et al. (age range 15–90 years) (dos Santos Pereira et al. 2011). However, the observed mean age of male cases was higher than that of females (*p* = 0.312), which contrasts with de Azevedo et al. (2021). The male‐to‐female ratio in our study (2.28:1) is comparable to Mahmood et al. (1.5:1) (Mahmood et al. 2022) and Tang et al. (1.07:1) (Tang et al. 2020) but contrasts with de Azevedo et al. (1:1.27) (de Azevedo et al. 2021) and dos Santos Pereira et al. (0.7:1) (dos Santos Pereira et al. 2011) who reported higher ratios of females to males.

Our study found a statistically significant difference between age groups and the risk of lesions, while de Azevedo et al. (2021) found a significant relationship between age groups and grades of OED. Older age and female gender have been associated with an increased rate of progression to OSCC (de Azevedo et al. 2021; Kujan et al. 2006; Saleem Khan et al. 2021). Old age is a known predictive risk factor for the development of OSCC, and numerous reports indicate that women, although less likely to develop OPMDs, are more prone to malignant transformation compared to men (Speight et al. 2018; Warnakulasuriya and Ariyawardana 2016).

### 4.4 Comparison of WHO and Binary Grading Systems

A statistically significant difference was observed between the WHO and the binary grading system of OED, which aligns with the study by de Azevedo et al. (2021). In our study, moderate dysplasia was the most common grade, followed by mild dysplasia, with fewer cases of severe dysplasia. These findings are similar to those of dos Santos Pereira et al. (2011), who reported more cases of mild dysplasia compared to moderate dysplasia. Severe dysplasia was also reported less frequently than other forms of OED by other authors (Aittiwarapoj et al. 2019; Pires et al. 2020; Mao et al. 2020). OED is considered a significant prognostic factor for progression to oral epithelial malignancy (Speight et al. 2018).

Our study observed a higher number of oral leukoplakia cases classified as low‐risk lesions compared to high‐risk lesions. None of the oral lichen planus cases fell into the high‐risk dysplasia, whereas most cases of speckled leukoplakia were categorized as high‐risk dysplasia. Histologically, most cases of moderate dysplasia and all cases of severe dysplasia were classified as high‐risk dysplasia. These observations are consistent with the literature regarding the risk of malignant transformation in OPMD lesions with varying degrees of dysplasia (Ganesh et al. 2018; Nagata et al. 2018). In our study, 54.9% of cases were classified as high‐risk dysplasia and 45.1% as low‐risk dysplasia, aligning with Câmara et al. (2016) but contrasting with Kujan et al. (2006) and Shubhasini et al. (2017). OED is a significant prognostic factor for progression to oral epithelial malignancy (Diajil et al. 2013).

Not every potentially malignant lesion will develop into oral epithelial malignancy, and OSCC may arise in lesions even without OED (Khan et al. 2023). This is true for cases of proliferative verrucous leukoplakia (El‐Naggar et al. 2017). The binary system further classifies OPMDs with OED into low‐risk and high‐risk categories (Kujan et al. 2006). Liu et al. (2011) reported that oral epithelial malignancy can also arise in low‐risk OPMDs but observed that high‐risk OED has a significantly higher potential (2.78‐fold) for malignant transformation compared to low‐risk OPMDs and labeled high‐risk dysplasia as an independent risk factor for malignant transformation in potentially malignant epithelial lesions. The present study also found a statistically significant relationship between the binary grading system of OED and the WHO grading system, in accordance with the findings of Câmara et al. (2016).

### 4.5 Subepithelial Lymphoplasmacytic Infiltrate

We observed subepithelial inflammatory infiltrate in cases of OPMDs, with most lesions expressing LPI. A previously published study described the clinicopathological characteristics of OPMDs with dysplasia and reported the presence of subepithelial inflammatory infiltrate (Khan et al. 2023). However, that study did not evaluate the graded patterns of LPI or examine their association with the binary grading system of OED. The present study expands upon those findings by introducing a structured subgrading system for LPI and assessing its clinicopathological correlations with both the WHO and binary grading systems of OED.

The biological basis of LPI in OPMDs may be related to the complex interaction between inflammatory and immune cells within the epithelial–stromal microenvironment. The LPI comprises predominantly lymphocytes and plasma cells, although other immune‐cell populations may also contribute to the local inflammatory response. Previous studies have demonstrated increased infiltration of CD4+ and CD8+ T cells and B cells with progression of oral epithelial lesions, suggesting an active immune response during oral carcinogenesis (Surendran et al. 2022). More recent studies have shown that high‐grade OED may contain increased lymphocyte infiltration together with immune‐suppressive features, including regulatory T cells and increased expression of immune checkpoint molecules such as PD‐1 and PD‐L1 (Flores‐Hidalgo et al. 2024; Sun et al. 2016). These findings suggest that the inflammatory infiltrate may have a dual role, with immune surveillance occurring alongside mechanisms that can suppress effective antitumor immunity. In addition, cytokines and other inflammatory mediators, including IL‐10 and TGF‐β, may contribute to the development of an immunosuppressive microenvironment and facilitate persistence or progression of dysplastic epithelial cells (Sun et al. 2016; Shaikh et al. 2024). Therefore, increasing LPI may reflect changes in the local immune microenvironment associated with OED severity. However, because the present study assessed LPI morphologically and did not characterize individual immune‐cell populations or molecular mediators by immunohistochemistry or molecular methods, the specific cellular and molecular mechanisms underlying the proposed LPI grades remain to be established.

Inspired by the work of Halter and Dellon (1983) and Bryne et al. (1992), we assessed and reported the subgrading of LPI. This variable, reflecting the immunological response of host tissue to OSCC lesions, has not been previously reported in the context of OPMDs with OED. Halter and Dellon (1983) evaluated LPI subgrades in benign, borderline, and malignant skin lesions, while Bryne et al. (1992) introduced a multifactorial grading system reflecting host tissue response. Our study found that most cases had a moderate inflammatory infiltrate, followed by slight and marked infiltrates.

The present study reveals that the risk of lesion is related to LPI and its subgrades, highlighting their potential role in the malignant transformation of OPMDs with OED. This finding should, however, be interpreted cautiously because the present study is cross‐sectional and does not include follow‐up data for malignant transformation.

## 5 Strengths

This study offers several notable strengths. It is the first to assess the relationship between two distinct grading systems for OED—the WHO grading system and the binary grading system—and LPI and its newly proposed grades. By integrating these systems and exploring their interactions with subepithelial LPI, this research provides valuable insights into the clinicopathological characteristics of OPMDs with OED, contributing significantly to the existing literature.

## 6 Limitations

The study has several limitations. Notably, it does not include cases of oral submucous fibrosis, which limits the generalizability of the findings to this specific condition. Another limitation is the absence of follow‐up data to monitor the malignant transformation of OED cases, particularly high‐risk dysplastic lesions. Also, the inclusion of OLP in the study is a limitation, as OLP often presents with dense LPI at the epithelia–mesenchymal junction, which may not reflect the broader spectrum of newly proposed LPI grades. Additionally, as a nonprobability sampling technique, the study participants may not be a perfect representative of the population of Peshawar, Pakistan. Furthermore, the proposed grading of LPI has not yet undergone external validation and should be interpreted cautiously until confirmed by larger prospective studies. Finally, non‐causal design, residual confounding, and information bias, specifically recall bias among the tobacco users, also add to the list of limitations.

## 7 Conclusion

Numerous clinical and histopathological parameters can predict the prognosis of OPMDs. Our study found a significant relationship between the grades of OED and subepithelial LPI. The risk of oral potentially malignant lesions was significantly related to the clinical subtypes of OPMDs, histological grades of OED, subepithelial LPI, and various grades of subepithelial LPI. The degree of LPI was related to the severity of OED lesions, highlighting the significant relationship between the risk of an OPMD lesion and subepithelial LPI. The findings demonstrate a significant association between the degree of LPI and the severity of OED. These observations suggest that subepithelial LPI may reflect underlying microenvironmental changes associated with dysplasia severity. However, longitudinal studies with follow‐up data are required to determine whether LPI can serve as a true prognostic predictor of malignant transformation.

## 8 Recommendations and Future Directions

To enhance understanding and improve clinical outcomes, we recommend conducting further studies that include a variety of OPMD entities with detailed follow‐up data to assess malignant transformation rates. This will provide a more comprehensive view of the progression and risk factors associated with these lesions, ultimately aiding in more effective management and treatment strategies.

## Author Contributions

Abbas Saleem Khan contributed to the conceptualization and design of the study, histopathological evaluation of the specimens, supervision of the research process, and critical revision of the manuscript. Shahzad Ahmad contributed to the study design, data interpretation, and manuscript preparation. Hina Shabir participated in histopathological assessment, data collection, and contributed to manuscript drafting. Shazia Iqbal contributed to data interpretation, literature review, and critical revision of the manuscript for important intellectual content. Tehmina Naushin participated in histopathological evaluation, observer calibration, and data verification. Muhammad Nadeem Baig contributed to data analysis, interpretation of results, and manuscript editing. Aneela Bashir assisted in data collection, literature review, and manuscript preparation. Maria Hanif contributed to data compilation, literature search, and manuscript editing. Mohammed Saad Alqarni contributed to manuscript review, interpretation of findings, and critical revision of the manuscript. Rakhi Issrani contributed to statistical analysis, interpretation of data, and manuscript editing. All authors read and approved the final version of the manuscript.

## Funding

The authors have nothing to report.

## Ethics Statement

Ethical approval for this study was obtained from the Institutional Review Board of Prime Foundation Pakistan under Approval No.: Prime/IRB/2021‐287, Dated March 10, 2021.

## Consent

The authors consent to the publication of this work.

## Conflicts of Interest

The authors declare no conflicts of interest.

## Data Availability

The data for this study are available upon request from the corresponding authors.
